# Cyborg and Bionic Systems: Signposting the Future

**DOI:** 10.34133/2020/1310389

**Published:** 2020-09-28

**Authors:** Toshio Fukuda

**Affiliations:** Beijing Institute of Technology, China

*Cyborg and Bionic Systems* provides a much-needed forum for presenting research outcomes and insights in various fields concerning living-robotics hybrid systems. The research efforts within these fields are aimed at understanding, mimicking, and recapitulating natural principles and mechanisms of life, which have inspired numerous inventions and propelled technology advancements throughout history.

From cardiac pacemakers to artificial cochlea, a range of biomedical applications of cyborg and bionic system technologies has benefited humans. Artificial organs, exoskeletons with neural interfacing, and brain-machine interfaces (BMIs) for neurological recovery have been applied to replace or repair lost functions. An example of inspiring achievements and the potential of cyborg and bionic system technologies to restore human capabilities is CYBATHLON, a championship for physically challenged people using assistive technologies which began in 2016. These technologies are closely coupled with the “embodiment” issues which are essentially concerned with the psychological and sensing aspects of prosthetic arms and legs and even extra artificial limbs to augment our body for carrying out various tasks, a concept championed by a pioneering team led by Professor Harry Asada at MIT through a technology aptly named Supernumerary Robotic Limbs (SRL).

To decipher the mysteries of life and to address challenges in pivotal industries such as pharmaceuticals and medical devices, the mimicry of life has spurred ingenious and ambitious innovations. Bionic Humanoids, a project initiated by Japanese researchers, strives to build realistic human simulation models for development and tests of medical devices and surgery procedures. Lab-on-chips that emulate human organs have been developed and advanced with the aim of improving the current practices in drug discovery, drug testing, and disease modelling. Research teams around the world are advancing technologies that simulate the human body on tissue, organ, and even biocell levels. A team at Wyss Institute of Harvard University engineered an automated instrument, called the Interrogator, which is able to link and culture multiple organs-on-chips to mimic a multiorgan system [1].

Advancements in micro-/nanotechnology, biosensors, and biomaterials have spawned novel and promising applications. Sensor systems that can be comfortably mounted to the skin to offer continuous monitoring of COVID-19-related systems have been tested, though results of these studies have yet to be revealed [2]. A team at the Chinese Academy of Sciences (CAS) developed a DNA nanorobot to deliver therapeutic thrombin at the tumor site, a promising method for cancer therapy [3].

Recent advancements in cyborg and bionic system technologies have also triggered controversies. Notably, an AI-based method for outputting reconfigurable organisms was presented in 2019 as a result of crossdisciplinary collaboration [4]. The study has spurred questions about the ontological nature of such a novel life form, the risks of the applications that such research opens up, and wider moral and ethical concerns [5]. Advancements in cyborg and bionic technologies will continue to engage scientists, bioethics, and general stakeholders in meaningful dialogues, which the journal intends to present in addition to its research-focused articles.

I am honored to serve as the founding Editor-in-Chief, and I am grateful to have a highly qualified group of editors to join me in building *Cyborg and Bionic Systems* into a leading journal for the research community at the intersection of robotics, neurosciences, neuroengineering, and bioengineering.

*Toshio Fukuda*
